# The Mitochondrial Genomes of 18 New Pleurosticti (Coleoptera: Scarabaeidae) Exhibit a Novel trnQ-NCR-trnI-trnM Gene Rearrangement and Clarify Phylogenetic Relationships of Subfamilies within Scarabaeidae

**DOI:** 10.3390/insects12111025

**Published:** 2021-11-14

**Authors:** Sam Pedro Galilee Ayivi, Yao Tong, Kenneth B. Storey, Dan-Na Yu, Jia-Yong Zhang

**Affiliations:** 1 Department of Biology, College of Chemistry and Life Science, Zhejiang Normal University, Jinhua 321004, China; paydrov17@gmail.com (S.P.G.A.); tyty9901@163.com (Y.T.); 2 Department of Biology, Carleton University, Ottawa, ON K1S5B6, Canada; KennethStorey@cunet.carleton.ca; 3 Key Lab of Wildlife Biotechnology, Conservation and Utilization of Zhejiang Province, Zhejiang Normal University, Jinhua 321004, China

**Keywords:** Scarabaeidae, phytophagous, mitochondrial genome, gene rearrangement, non-coding region (NCR), phylogeny

## Abstract

**Simple Summary:**

The family Scarabaeidae is one of the largest families in the insect order Coleoptera and is comprised of two quasi-systematics groups, Pleurosticti and Laparosticti. Pleurosticti is an economically important scarab group comprising about 20,000 species, the majority of which are phytophagous. Despite the innumerable studies based on ecological, molecular, and morphological characteristics, their taxonomy is still unclear and subject to many scientific hypotheses. The mitochondrial (mt) genome can provide tangible information to resolve the phylogenetic relationships within the family Scarabaeidae. However, the available mt genomes of Scarabaeidae in GenBank are underrepresented. Thus, we sequenced and analyzed 18 new phytophagous Scarabaeidae mitochondrial genomes from two subfamilies, Cetoniinae and Dynastinae, to conduct phylogenetic analyses within Scarabaeidae. This study contributes to increasing our knowledge about phytophagous Scarabaeidae.

**Abstract:**

The availability of next-generation sequencing (NGS) in recent years has facilitated a revolution in the availability of mitochondrial (mt) genome sequences. The mt genome is a powerful tool for comparative studies and resolving the phylogenetic relationships among insect lineages. The mt genomes of phytophagous scarabs of the subfamilies Cetoniinae and Dynastinae were under-represented in GenBank. Previous research found that the subfamily Rutelinae was recovered as a paraphyletic group because the few representatives of the subfamily Dynastinae clustered into Rutelinae, but the subfamily position of Dynastinae was still unclear. In the present study, we sequenced 18 mt genomes from Dynastinae and Cetoniinae using next-generation sequencing (NGS) to re-assess the phylogenetic relationships within Scarabaeidae. All sequenced mt genomes contained 37 sets of genes (13 protein-coding genes, 22 tRNAs, and two ribosomal RNAs), with one long control region, but the gene order was not the same between Cetoniinae and Dynastinae species. All mt genomes of Dynastinae species showed the same gene rearrangement of trnQ-NCR-trnI-trnM, whereas all mt genomes of Cetoniinae species showed the ancestral insect gene order of trnI-trnQ-trnM. Phylogenetic analyses (IQ-tree and MrBayes) were conducted using 13 protein-coding genes based on nucleotide and amino acid datasets. In the ML and BI trees, we recovered the monophyly of Rutelinae, Cetoniinae, Dynastinae, and Sericinae, and the non-monophyly of Melolonthinae. Cetoniinae was shown to be a sister clade to (Dynastinae + Rutelinae).

## 1. Introduction

The insect order Coleoptera (>400,000 species), also known as the most largest and diverse group of beetles on Earth [1,2], represents one-quarter of all known animal species [3]. The Suborder Polyphaga with approximately 300,000 species is the largest group within the four Coleoptera suborders, including Archostemata, Adephaga, Myxophaga, and Polyphaga. Polyphaga beetles are distributed worldwide and include the groups with the largest length bodies (>19 cm, e.g., *Dynastes hercules*) and the heaviest (>200 g, e.g., *Megosoma actaeon)* beetles that belong to the Family Scarabaeidae [4,5].

The family Scarabaeidae, with over 30,000 large and diversified species distributed worldwide, was divided into coprophagous and phytophagous groups according to their feeding habits [6,7]. The coprophagous species of Scarabaeidae are economically and ecologically important and include the two subfamilies (Scarabaeinae and Aphodiinae) [8,9]. Also called dung beetles, the coprophagous beetles mostly use dung as food, and the subfamily Scarabaeinae were qualified as the true dung beetles among this group [10]. The phytophagous species of Scarabaeidae, commonly known as Pleurosticti [11], comprise about 20,000 species and represent approximately 70% of scarab beetles [7,12]; most of these species feed on vegetation [13]. This group is represented by the subfamily Dynastinae (1500 species), Cetoniinae (3300 species), Rutelinae (4000 species), Melolonthinae (11,000 species) [12], and Sericinae [14], the latter being considered in some taxonomies as the tribe Sericini of the subfamily Melolonthinae [15,16,17]. Hence, Pleurosticti scarabs are shown as a group of four major subfamilies in some studies. 

For this reason, the taxonomy and phylogeny of Sericinae and Melolonthinae have been the subject of scientific debate during past years [6,18,19,20,21]. Recent research papers propose to elevate the tribe Sericini to a subfamily rank [6,14], and at the same time it was recommended to collect new data on Dynastinae to resolve its phylogenetic relationship with Rutelinae. In addition, the monophyly of Cetoniinae, Dynastinae, and Rutelinae and their phylogenetic relationships need to be clarified due to their rich diversity. Many types of research based on feeding habits, morphology, sex pheromones (chemical ecology), and genomes [6,7,12,22,23,24,25] have been conducted in attempts to resolve the phylogenetic relationships but have still not been conclusive. Moreover, some subfamilies (Ex: Dynastinae-Cetoniinae, Rutelinae-Melolonthinae) share the same characteristics (feeding, morphology, habitat). The species of Dynastinae and Cetoniinae have great economic importance due to their activity in different ecosystems. Dynastinae species, also called rhinoceros beetles because of the spectacular horns of the males, share some characteristics with the subfamily Cetoniinae species, such as rotting wood [22] and the presence of a setal field on the external basal surface of the first lamella [7]. In addition, some Dynastinae and Cetoniinae grubs feed in soil humus or litter [24]. 

The mitochondrial (mt) genome is an ideal molecular marker that provides comprehensive genetic information to study population origin, evolution and phylogeny relationships [26,27]. The mt genome in insects is small with a circular DNA structure and comprising an total 22 tRNAs, 13 protein-coding genes (PCGs), two ribosomal RNAs, and a single major non-coding region (called as D-loop region or control region or A + T rich region) [28]. According to the size of the control region and different gene lengths, the insect mitochondrial genomes are generally 14–20 kb in length [27]. Mitochondrial genomes with characteristics such as rare sequence recombination, maternal inheritance, gene conservation, and high evolution rate represent an essential tool for comparative studies [29,30]. A comparative study based on gene arrangement, length variation, atypical start codon, and base composition bias permits analysis of the molecular differences among species to reconstruct taxonomy [31]. Mitochondrial genome sequencing is facilitated by the progress of next-generation sequencing (NGS). However, to date, the number of mt genomes among phytophagous scarab beetles is underrepresented in GenBank (accessed on 4 September 2021), especially for the subfamily Dynastinae (only three complete mt genome sequences: one of *Eophileurus chinensis* and two of *Oryctes rhinoceros*) and Cetoniinae (five mt genomes: two of *Osmoderma opicum* and three of *Protaetia brevitarsis*) compared with the rest of the subfamilies of the family Scarabaeidae. However, these two groups show some differences in mt gene arrangement, in particular, the gene rearrangement of trnQ-trnI-trnM in Dynastinae [18,19]. We reanalyzed all species of Rutelinae and Melolonthinae, Scarabaeinae and Aphodiinae in NCBI to determine if mt gene rearrangements were present in other groups of the family Scarabaeidae, but no gene rearrangement in these subfamilies were found.

In this study, we sequenced, annotated, and analyzed 18 new mt genomes of Dynastinae and Cetoniinae (nine species or sub-species from each subfamily) to improve the representation of these species in GenBank and allow a comparative mitogenomic study based on genome size, nucleotide composition, gene rearrangement, and start codon between the two subfamilies. Furthermore, with the aim to describe the mitochondrial genome structure in these species and establish their phylogenetic relationships within the family Scarabaeidae, we additionally added mt genomes of 34 beetles downloaded from NCBI GenBank that belong to all the subfamilies of Scarabaeidae (Dynastinae, Cetoniinae, Rutelinae, Melolonthinae, Sericinae, Aphodiinae, and Scarabaeinae).

## 2. Materials and Methods

### 2.1. Taxon Sampling and Mitogenome DNA Sequencing

All specimens of nine species from subfamily Cetoniinae, and nine species or subspecies from subfamily Dynastinae used in this study were identified based on external morphology features by Dr. J.Y Zhang and COX1 gene DNA barcoding was blasted in NCBI and BOLD. All specimens are preserved in the Museum of Zoology, Zhejiang Normal University, China. Total genomic DNA of each species was extracted from thoracic muscle using Ezup Column Animal Genomic DNA Purification Kit (Sangon Biotech Company, Shanghai, China). The DNA of all specimens was sent to BGI Tech. Inc. (Shenzhen, China) for Next Generation Sequencing on the Illumina MiSeq Platform using the shotgun method. Raw paired reads (Original data) were subjected to fastQC [32,33] for quality control check and trim. The clean data were used for assembling the mitochondrial genome of each species through NOVOPlasty [34], with *Oryctes rhinoceros* (Dynastinae: MT457815) and *Protaetia brevitarsis* (Cetoniinae: KC775706) as each group (subfamilies Cetoniinae and Dynastinae species) references sequences (seed sequences).

### 2.2. Gene Annotation and Sequence Analyses

The eighteen assembled mitochondrial genomes were annotated using the Mitos2 web server [35] with the parameter 5 settings for invertebrate Genetic Code. The position and secondary structure of tRNAs were confirmed by the tRNAScan-SE online search Server [36]. The 12S and 16S rRNAs were further determined by comparing homologous genes from other Cetoniinae and Dynastinae species mentioned previously using MEGA 7.0 [37]. The thirteen PCGs of all species were identified as open reading frames based on the invertebrate mitochondrial genetic code. PCGs without canonical start and stop codons were further adjusted after translation using MEGA 7.0. The tandem repeats in the control region were predicted using Tandem Repeats Finder online web server program (https://tandem.bu.edu/trf/trf.basic.submit.html, accessed on 9 November 2021) [38].

The circular mitochondrial maps of Cetoniinae and Dynastinae species were draw using CG View online Server [39]. Nucleotide composition and composition skewness were determined by Mega 7.0 and the formulae AT skew = (A − T)/(A + T) and GC skew = (G − C)/(G + C), respectively. Codon usage and relative synonymous codon usage (RSCU) were analyzed using PhyloSuite V.1.2.2 [40] and graphically drawn by ggplot2 packaging in R [41]. 

### 2.3. Phylogenetic Analyses

For phylogenetic analyses, we combined 18 mt genomes in this study with 34 mt genomes downloaded from NCBI, including sequences from Cetoniinae (2), Dynastinae (2), Rutelinae (4), Melolonthinae (7), Sericinae (2), Aphodiinae (1), and Scarabaeinae (12) of Scarabaeidae, and four outgroups species belonging to Lucanidae (2), Trogidae (1), and Glaresidae (1) (Table 1).

Phylogenetic analyses were conducted by MrBayes and IQ-tree using all the nucleotide and amino acids extracted with PhyloSuite V1.2.2. To prepare the multiple sequence alignment for our analyses, we first translated the nucleotide sequences of 13 PCGs into amino acids (AA) in Mega 7.0 software under the invertebrate mitochondrial genetic code after checking the order generated by PhyloSuite V.1.2.2 software and then aligned with muscle implemented in Mega 7.0. After removing the stop codons and gaps manually, we saved the data to construct the dataset of amino acids. Finally, each amino acid was back-translated into nucleotide sequences to construct the nucleotide dataset of PCGs. All the aligned nucleotide datasets and amino acid datasets were concatenated using Mesquite software [51]. PartitionFinder version 2.2.1 was used to select the best partitioning scheme and substitution model for Bayesian inference (BI) and maximum likelihood (ML) analysis. The best-fit substitution results of nucleotide and amino acid datasets are shown in Supplementary Table S1. The heterogeneity of sequence divergence among taxa using the nucleotides and amino acids datasets were tested using AliGROOVE [52] with the default sliding window size.

BI and ML analyses were performed to construct Phylogenetic trees using MrBayes [53] and IQ-tree [54], respectively. For BI analysis with MrBayes, GTR + I + G and MTART + I + G were the best-fit partition models for the nucleotides and amino acids datasets, respectively. MrBayes, under default settings and 5 × 10^6^ Markov Chain Monte Carlo (MCMC) generations with sampling every 1000 generations, was used for phylogenetic tree construction. The first 25% of sampled data were discarded as burn-in, and the average standard deviation of split frequencies below 0.01 was considered to reach convergence. The best-fit partition model for ML analysis using IQ-tree was also GTR + I + G and MTART + I + G for the nucleotides and amino acids datasets, respectively. ML phylogenetic analysis was performed with the evaluation of branch support for each node under 1000 ultrafast replication.

## 3. Results

### 3.1. General Features of mt Genomes

**Figure 1:**
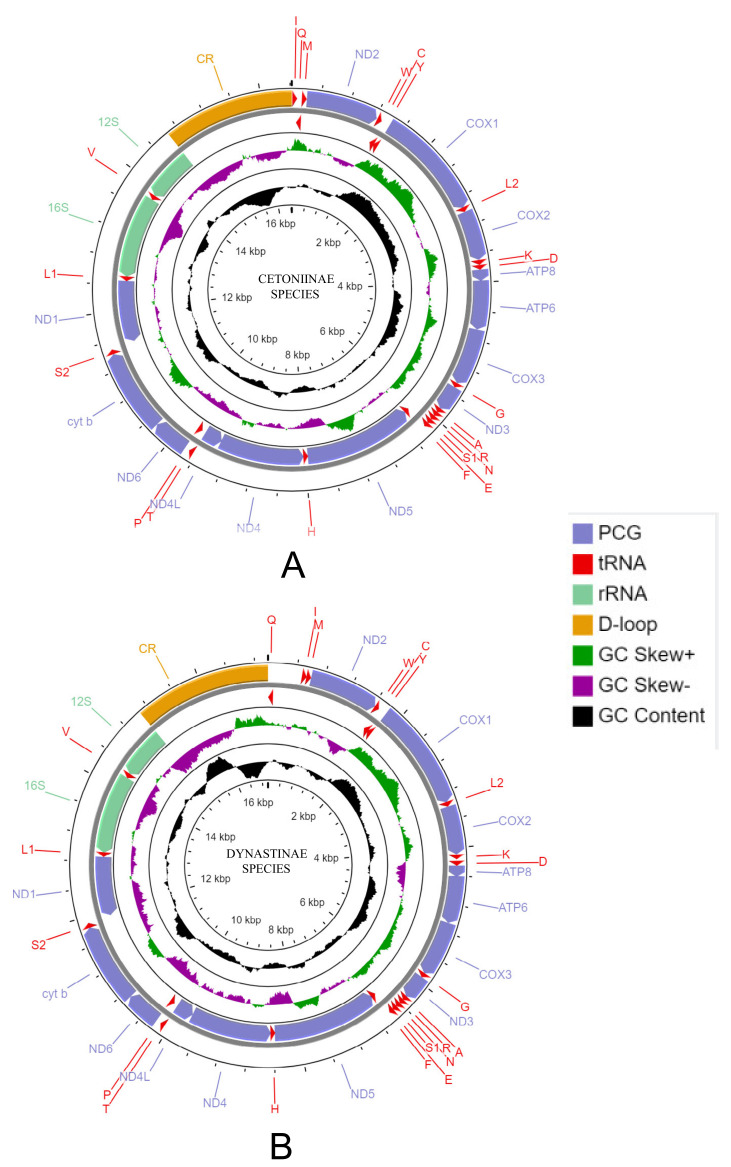
Representative mitochondrial genome maps of Cetoniinae (**A**) and Dynastinae (**B**) species. The first circle shows the gene map (PCGs, rRNAs, tRNAs and the AT-rich region) and the genes outside the map are coded on the majority strand (J-strand) whereas the genes inside the map are coded on the minority strand (N-strand). The second circle shows the GC content and the third shows the GC skew. GC content and GC skew are plotted as the deviation from the average value of the entire sequence.

Nine newly sequenced mt genomes of Cetoniinae species were used for our comparative study: *Protaetia speciosa jousselini, Cyprolais quadrimaculata, Dicronorhina derbyana, Eudicella euthalia oweni, E. smithi, Goliathus goliatus, Jumnos ruckeri, Mecynorhina polyphemus, M. torquata ugandensis*. All nine species showed the typical circular double-stranded molecules (Figure 1A) with lengths ranging from 16,422 bp *(M. polyphemus*) to 19,468 bp (*Jumnos ruckeri*) (Figure S1). Each mt genome contained a putative non-coding region and the complete set of 37 genes comprised of two ribosomal RNA genes (rRNAs), 22 transfer RNAs (tRNAs), and 13 protein-coding genes (PCGs) (Table S2). Among the 37 genes, nine PCGs and 14 tRNAs were encoded in the majority strand (J-strand), whereas the other four PCGs, eight tRNAs, and two rRNAs were located on the minority strand (N-strand). The tRNAs of Cetoniinae species presented the classical cloverleaf secondary structure. The A-T content of the whole mitogenome and PCGs were calculated, and the results ranged from 71.8% *(J. ruckeri*) to 75.6% (*M. torquata ugandensis*), and from 71.2% (*Jumnos ruckeri*) to 73.6% (*M. torquata ugandensis*, *G. goliatus*), respectively. The A-T skew and G-C skew of the whole mt genome were positive and negative, respectively (Supplementary Table S3). At the same time, A-T and G-C skew values were both negative in PCGs (−), but negative and positive in PCGs (+), respectively (Supplementary Table S4). The numbers of overlapping regions present in the mt genome vary from one species to another, and their numbers range from 10 to 14, with sizes comprising between 1 bp and 8 bp. Except *Eudicella euthalia oweni* and *E. smithi*, the most extended overlapping region of 8 bp was located in all species between trnW and trnC.

The second group in our comparative analysis was constituted of nine newly sequenced Dynastinae species and sub-species that were: *Chalcosoma caucasus caucasus*, *Dynastes hercules hercules*, *Megasoma elephas elephas*, *M. mars, O. nasicornis*, *Xylotrupes beckeri intermedius*, *X. gideon beckeri*, *X.* *g. siamensis*, *X.* *g. sumatrensis.* The length of their whole mt genome ranged from 16,785 bp (*M.* *e. elephas*) to 20,396 bp (*O. nasicornis)* with A-T content ranging from 67.1% (*D.* *h. hercules*) to 77.1% (*X.* *g. siamensis*) (Supplementary Table S3). The mt genomes of Dynastinae species were also composed of 37 genes described previously, and these genes shared the same features (strand location and structure) as Cetoniinae species (Figure 1B and Figure S1, Tables S2 and S3). The A-T content of PCGs ranged from 66.3% (*D.* *h. hercules*) to 75.5% (*X.* *g. siamensis*) (Supplementary Table S4). The mt genomes of all Dynastinae species used in this study generally showed 10 overlapping regions, except *M.* *e. elephas* and *M. mars* that contained 11 overlapping regions. Overlapping regions ranged from 1 bp to 8 bp with the most extensive overlap region of 8 bp being situated in all species and sub-species between trnW and trnC.

### 3.2. Gene Arrangement and Non-Coding Region

The gene order in the 18 new species and sub-species of subfamilies Cetoniinae and Dynastinae was different. We found that the 37 sets of genes in Cetoniinae species respected the ancestral insect gene order pattern. By contrast, all Dynastinae species showed a minor gene order rearrangement between tRNA I and Q, including the insertion of a non-coding region resulting in a trnQ-NCR-trnI-trnM gene cluster that was markedly different from the ancestral trnI-trnQ-trnM gene cluster. The length of NCR in *C. c. caucasus, D. h. hercules, O. nasicornis, X. b. intermedius, X. g. beckeri, X. g. siamensis,* and *X. g. sumatrensis* ranged from 56 bp to 71 bp, whereas the length of NCR in *M. e. elephas* and *M. mars* was 412 bp (Supplementary Table S5). According to the classification of gene movements, this novel gene order in the subfamily Dynastinae qualifies as a gene translocation event between trnI and trnQ where these two genes swapped positions [26,27,55,56]. The NCR between trnQ and trnI generated by the gene rearrangement can be divided into two types of NCR1 (<100 bp) and NCR2 (>400 bp).

The non-coding region in phytophagous Scarabaeidae was relatively conserved. Six intergenic spaces (IGS) were conserved among the Cetoniinae species except for *Jumnos ruckeri* that showed five intergenic spaces and *M. torquata ugandensis* and *Goliathus goliatus* with seven IGS. The longest IGS (16–21 bp) in Cetoniinae was located between trnS2 and ND1, and all contained the motif of hexanucleotides TACTAA (Supplementary Figure S2A). The mt genomes of Dynastinae species also generally showed six IGS except *Chalcosoma caucasus caucasus,* *D. h. hercules, M. e. elephas, M. mars* that contained seven, eight, eight, and eight IGS, respectively. Unexpectedly, we found that *D. h. hercules, M. e. elephas,* and *M. mars* showed a particularly long IGS of 20 bp, 23 bp, and 23 bp, respectively, between trnK and trnD, and all of these contained homopolymeric stretches of cytosine (C-stretches of 11 bp) (Supplementary Figure S2B), apart from the common IGS located between trnS2 and ND1 with a length that ranges from 17 bp to 24 bp in Dynastinae, highlighting the presence of the pentanucleotide motif of TACTA (Supplementary Figure S2C). Furthermore, in Cetoniinae and Dynastinae, we generally found the 5 bp (TACTA) consensus motif, which is present in most of Coleoptera and Lepidoptera [57,58,59,60,61].

Beyond the NCR or IGS spread over the mt genome, an A-T rich region (control region) occurred in all species and was located between 12S RNA and trnI in Cetoniinae and between 12S RNA and trnQ in Dynastinae. The A-T rich region can be divided into two parts, the region of tandem repeated sequences and a subregion of higher A + T content. In Cetoniinae, the size of the control region is variable, ranging from 1776 bp (*M. Polyphemus)* to 4828 bp (*J. ruckeri)* with A-T content ranging from 70.22% (*J. ruckeri)* to 84.74% (*M. Polyphemus)*. Species of the subfamily Dynastinae showed a control region (CR) ranging from 1723 bp (*M. e. elephas*) to 5675 bp (*O. nasicornis*) with 66.11% (*D. h. hercules*) to 81.19% (*X. g. siamensis*) of A-T content (Supplementary Tables S6 and S7). In Dynastinae, we found some translatable nucleotide sequences in two or three copies in the repeat sequences region downstream of 12S RNA, and also in NCR2 of *Megosoma* species. Some of them contain open reading frames but did not have any significant or corresponding BLAST hits in NCBI [19]. In addition, we noted that the longest control region in the subfamily Cetoniinae and the Dynastinae presented an A + T content less than 75%, being 70.22% in *J. ruckeri* and 69.97% in *O. nasicornis*.

### 3.3. Protein-Coding Genes and Codon Usage

The total size of the 13 PCGs of Cetoniinae species was 11,136 bp for *Protaetia speciosa jousselini*, *Goliathus goliatus*, *Mecynorhina polyphemus*, *M. t. ugandensis*, and 11,139 bp for *Cyprolais quadrimaculata*, *Dicronorhina derbyana*, *Eudicella euthalia oweni*, *E. smithi*, and *Jumnos ruckeri* with negative AT skew and GC skew. For Dynastinae species, the overall length of the 13 PCGs was 11,133 bp in *Chalcosoma caucasus caucasus*, *Megasoma elephas elephas*, *M. mars*, *Oryctes nasicornis*, *Dynastes hercules hercules*, and 11,124 bp in *Xylotrupes beckeri intermedius*, *X. g. beckeri*, *X. g. siamensis*, and *X. g. sumatrensis*. The nucleotide skew was also negative for AT and GC in Dynastinae. In both Cetoniinae and Dynastinae groups, nine PCGs (ATP6, ATP8, COX1, COX2, COX3, CYTB, NAD2, ND3, and ND6) were encoded by the J-strand, and the remaining four PCGs (ND1, ND4, ND4L, ND5) were translated from the N-strand. The shortest PCG was ATP8 (156 bp) in the two subfamilies, whereas the longest was ND5 with 1714 bp or 1716 bp, in all Dynastinae and Cetoniinae species, respectively (Supplementary Table S2).

In our newly sequenced Cetoniinae mt genomes, most of the PCGs started with the conventional start codon ATN (with N representing A, C, T, or G), except for COX1, that started with AAN (AAC or AAT) in all species and ND6 that started with GTC in *Cyprolais quadrimaculata*. Most PCGs terminated with the typical stop codons (TAA or TAG) except COX1, COX2 and COX3, that terminated with the incomplete stop codon T. For the Dynastinae mt genome, all 13 PCGs started with the typical ATN, whereas ND2 in *Dynastes hercules hercules* began with GTC. The three putative stop codons (TAA, TAG, T) found in Cetoniinae were also present in Dynastinae. The incomplete stop codon T was used in COX1, COX2, COX3, and ND5, whereas TAG was used in ND3 and CYTB, and TAA occurred in the remaining PCGs (Table 2). It has been proposed that the incomplete stop codon T is completed through post-transcriptional polyadenylation during the mRNA maturation process [61,62]. In addition, the atypical start codons, AAC and AAT, were hypothesized to be a correct start codon for COX1 [29,43,63].

Supplementary Figure S3 and Table S8 show the amino acid composition and graph of relative synonymous codon usage (RSCU) of species in the subfamilies Cetoniinae and Dynastinae. Except for the stop codon, the overall number of codons was 3702–3703 in the Cetoniinae species group but ranged from 3696 to 3702 in the Dynastinae species group. The four most frequently used codons were Leu2 (UUA), Ile (AUU), Phe (UUU), and Met (AUA) in both Cetoniinae and Dynastinae. Arg (CGC) was not used in two Dynastinae species, *Oryctes nasicornis* and *Xylotrupes beckeri intermedius,* whereas in Cetoniinae it was just *Eudicella smithi* species that missed the codon Ala (GCG).

### 3.4. Heterogeneous Sequences Divergence and Phylogenetic Analyses

**Figure 2:**
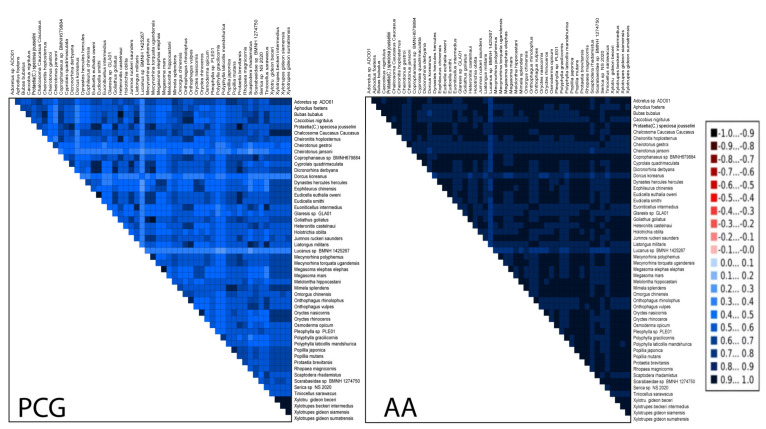
Heterogeneous sequence divergence within datasets of PCGs and AAs of all Scarabaeidae and outgroup species. The mean similarity score between sequences is represented by a colored square. AliGROOVE scores ranging from −1 (indicating great difference in rates from the remainder of the dataset) to +1 (indicating rates match in all other comparisons). Red coloring shows significant heterogeneity.

The obtained AliGROOVE matrixes (Figure 2) indicate positive similarity scores for the nucleotide and amino acid datasets in all taxon comparisons. The analyses revealed that the degree of heterogeneity of the concatenated nucleotide dataset was higher than the amino acid dataset. In particular, the pairwise sequence comparisons revealed high similarity among the family Scarabaeidae, as in Rutelinae, Dynastinae, Cetoniinae, and Sericinae species, whereas some Melolonthinae species showed high heterogeneity. This divergence in Melolonthinae indicated that some taxa of this group can be misplaced or cannot be robustly placed in the phylogenetic tree.

**Figure 3:**
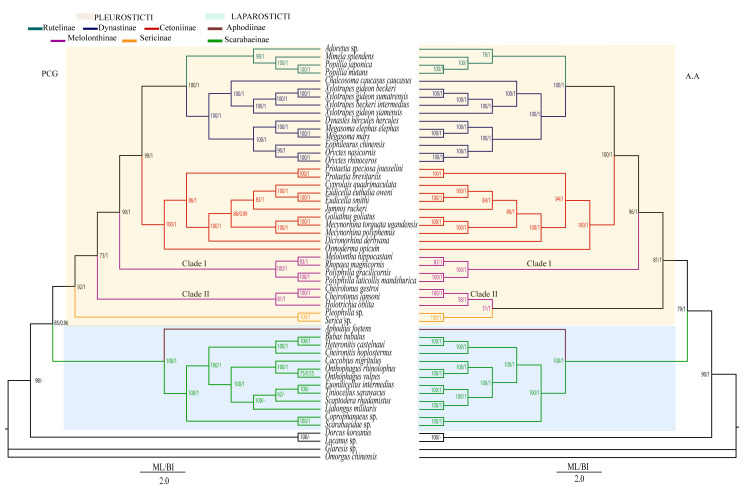
Phylogenetic relationships inferred by the ML and BI methods based on nucleotide (PCG, **Left**) and amino acids (AA, **Right**) datasets. Numbers on nodes are the posterior probabilities of BI (**Right**) and Bootstrap values of ML (**Left**). Different branch colors correspond to subfamily names.

We constructed four trees using BI and ML based on the concatenated nucleotide and AA sequences obtained from the total of 52 species belonging to the subfamilies Cetoniinae, Dynastinae, Rutelinae, Melolonthinae, Sericinae, Aphodiinae, Scarabaeinae, as well as the adding four outgroups species. The topological structure shown by BI (MrBayes) and ML (IQ-tree) using the nucleotide and the amino acid (AA) data was similar for each dataset. In BI and ML analyses, the two dataset trees differed from one another in Melolonthinae relationships. The two trees with AA datasets differed from that of the nucleotide dataset, which positioned Sericinae outside of the Melolonthinae group and placed it as the sister lineage to all other phytophagous species: (((((Rutelinae + Dynastinae) + Cetoniinae) + Melolonthinae I) + Melolonthinae II) + Sericinae). The topology of our trees using IQ-tree and MrBayes clearly showed the relationship between the two principal groups that constitute the family Scarabaeidae: Coprophagous and Phytophagous scarabs. Our 18 newly sequenced Cetoniinae and Dynastinae species were successfully placed in their corresponding subfamily clades in the trees with other Cetoniinae and Dynastinae species, retrieved from NCBI. In addition, the monophyly of Cetoniinae and Dynastinae was supported and the sister group relationship between (Rutelinae + Dynastinae) and Cetoniinae was strongly supported in all the analyses (BP = 99 and PP = 1). Melolonthinae was non-monophyletic in our analyses and can be regrouped into two small clades as follows: clade I ((*Polyphylla gracilicornis* + *Polyphylla laticollis mandshurica*) + (*Melolontha hippocastani* + *Rhopaea magnicornis*)) and clade II ((*Cheirotonus gestroi* + *Cheirotonus jansoni*) + *Holotrichia oblita*). Clade II Melolonthinae was nested within Sericinae in both BI and ML trees using the amino acid dataset, but was placed as a separate group based on the nucleotide dataset (Figure 3). The phytophagous and coprophagous Scarabaeidae clade was clearly shown as two different groups, and their phylogenetic relationship was coprophagous (Aphodiinae and Scarabaeinae) as the sister clade to phytophagous (Rutelinae, Dynastinae, Cetoniinae, Melolonthinae and Sericinae) (Figure 3). 

## 4. Discussion

### 4.1. General Features of mt Genomes in Cetoniinae and Dynastinae

In this study, we sequenced and presented 18 new scarab beetle species and sub-species belonging to the subfamilies Cetoniinae and Dynastinae to establish a comparative study and investigate the high-level relationships within Scarabaeidae based on the mitochondrial genome. Until now, there were too few papers that reported the complete mitochondrial genome of scarab beetles (especially among Pleurosticti) to be able to deeply analyze the genome structure, composition, and the phylogenetic relationships [18,19,29,43,45,64,65]. Our results show that the size of the mt genome varied widely among the examined species, ranging from 16,422 bp (*Mecynorhina polyphemus*) to 19,468 bp (*Jumnos ruckeri*) in Cetoniinae, and from 16,785 bp (*Megasoma elephas elephas)* to 20,396 bp (*Oryctes nasicornis)* in Dynastinae. These sizes are similar to others published for Scarabaeidae mitochondrial genomes. We found that the size of different PCGs, tRNAs, and rRNAs was largely conserved with just a slight variation among species, genus, and subfamily. This revealed that the size variation among species was mainly the result of significant differences in the size of the large control region (CR). The control region in Cetoniinae ranged from 1776 bp (*M. polyphemus)* to 4828 bp (*J. ruckeri*) and in Dynastinae from 1723 bp (*Megasoma* *e. elephas*) to 5675 bp (*O. nasicornis*)**.** In *P. brevitarsis* and *O. rhinoceros,* previous reported Cetoniinae and Dynastinae species, the CR was 5654 bp and 6204 bp, respectively [19,42]. Tandem repeats and high AT-rich region in the control region are observed in most scarab beetles and they represented a challenge for most commonly used methods for sequencing, including standard PCR sequencing with primers, Sanger, and short-read NGS. The difficulty encountered when sequencing the CR is due to its low G-C content, homopolymers, and tandem repeat sequences [28,59]. Except for the CR, the sequencing of the coding regions was unambiguous. In our sequences, we also detected the presence of homopolymeric stretches of cytosine (C-stretches) of 11 bp situated between trnK and trnD. These C-stretches were sequenced for the first time in beetle mitochondria by this study. Previous reports of homopolymeric stretches in beetles were common T-stretches in the Control Region, whereas C-stretches were observed in infectious laryngotracheitis virus and the human mitochondrial genome, all located principally in the control region [66,67]. The homopolymeric stretches in beetles are thought to signal and recognize proteins involved in replication initiation [68].

### 4.2. Gene Rearrangement

**Figure 4:**
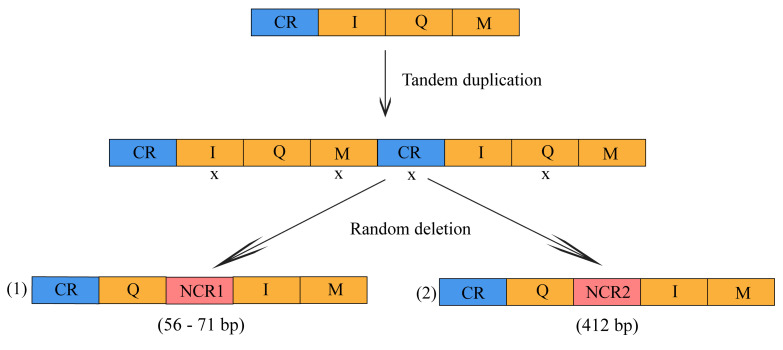
Gene rearrangement and locations of the generated non-coding regions between trnQ and trnI among Dynastinae species. The two non-coding regions (NCR) are colored in salmon (NCR1) and (NCR2). CR: control region; I, Q, M correspond to trnI, trnQ, trnM; (**1**) represent the gene rearrangement in: *Chalcosoma caucasus caucasus, Dynastes hercules hercules*, *Oryctes nasicornis*, *Xylotrupes beckeri intermedius*, *X. g. beckeri*, *X. g. siamensis*, *X. g. sumatrensis* species; and (**2**) represent: *Megasoma elephas elephas*, *M. mars* species gene rearrangement.

Gene rearrangement in the mt genome can be used as a phylogenetic marker due to the low rates of homoplasy because of the rare genomic changes, the conservation of secondary structures, and rare reversion of genes [26]. These characteristics make gene rearrangement a unique tool of synapomorphy and for analyzing evolutionary phylogenetic relationships among species [69]. In our sequenced Dynastinae species, we found gene rearrangement of trnQ-NCR-trnI-trnM. On the contrary, we did not find any gene rearrangement, duplication, or deletions among the newly sequenced Cetoniinae genomes, even when some previous complete mt genomes from the family Scarabaeidae were added from the NCBI. We conclude that there was no significant gene rearrangement in coprophagous or phytophagous scarab beetles except in the subfamily Dynastinae. A similar gene translocation has already been reported in the first sequenced Dynastinae subfamily species, *O. rhinoceros* [19], and the two recently reported Taiwanese rhinoceros beetles *O. rhinoceros* and *Eophileurus chinensis* [18]. Until now, there were only these three complete mitogenome sequences from the subfamily Dynastinae available in the NCBI and, as reported here, all of them shared this gene organization with our nine new sequenced mt genomes. Hence, this gene organization can be considered as characteristic or a synapomorphy for the subfamily Dynastinae, because gene rearrangements are uncommon and selectively neutral [56]. Gene position swapping between trnI and trnQ is also found in Hymenoptera and Hemiptera species [55,70,71] and was considered as one of the characteristic elements of these groups. From our study, we propose a tandem duplicate random-loss (TDRL) model to explain the gene rearrangement and the presence of the two different long noncoding regions (NCR1 < 100 bp, NCR2 = 412 bp) in the subfamily Dynastinae. Firstly, gene duplication occurred in the cluster CR-trnI-trnQ-trnM, considered as hotspot region for gene rearrangement in insect mitochondria. This generated the gene cluster (CR-trnI-trnQ-trnM-CR-trnI-trnQ-trnM). The duplication was followed by random loss and other mutations to become CR-trnQ-NCR1 (<100 bp) -trnI-trnM or CR-trnQ-NCR2 (>400 bp) -trnI-trnM (Figure 4). 

The gene rearrangement that placed the NCR between trnQ and trnI was only observed in the subfamily Dynastinae of Scarabaeidae, but was previously reported in Hemiptera: Aradidae and Reduviidae [70,71,72]. All the long NCRs showed some similarity with the control region (CR). The generated non-coding regions (NCR1 and NCR2) between trnQ and trnI ranged from 56 bp to 412 bp long. The long NCR2 of 412 bp was found only in *Megosoma*, especially in *M. mars* and *M. e. elephas* because the random deletion was not as extensive as in other species with NCR1. This is the first time that this kind of long non-coding region (>400 bp) was sequenced in the family Scarabaeidae. All these portions of sequences that are situated between trnQ and trnI qualified as non-coding regions because of their low similarity (<70%) with the genes present in the CR-trnI-trnQ-trnM region. For this reason, we proposed that these sequences of NCR underwent a series of mutations and evolved under relaxed selective pressure to become another degenerative control region. Unexpectedly, we found that the first 231 bp of the 412 bp long NCR in *Megasoma elephas elephas* and *M. mars* can be translated into an amino acid using the invertebrate mitochondrial genetic code, but they did not show any similarity with protein coding genes or amino acids available in the NCBI database. We speculated they could be non-coding RNAs generated from “mitochondrial dark matter” [19]. This situation was previously observed in seed beetles [73] and Dynastinae beetles [19] (*O. rhinorceros* control region). However, the characterization of these sequences needs further study using more transcriptomes.

### 4.3. Start Codons

Our analyses revealed that the start codons in protein-coding genes of Cetoniinae species differed from those of Dynastinae, especially in the COX1 gene. Four start codons, ATA, ATT, ATC, and ATG, are recognized as conventional or canonical start codons. For years, the COX1 start codon has been the subject of scientific discussion. The COX1 gene has been shown to start with some atypical start codons in many genomes. This problem was first encountered in the Drosophila mt genome [74,75], where ATAA was proposed as a start codon. AAT, AAC, TTG, CAA, CGA, AAA, and CTA have also been used as start codons in different species [31,76,77,78,79,80]. In our study, we proposed AAN (N replacing C or T) as a start codon for COX1 in all our newly sequenced Cetoniinae mitochondrial genomes, whereas in Dynastinae, we did not find any coding genes that started with AAN. In all species of the subfamily Cetoniinae, the possible canonical ATN start codon for the COX1 gene was situated at 34 bp downstream of trnT. To minimize intergenic space and avoid gene overlaps, because COX1 was demonstrated to not overlap with the upstream tRNA-Tyr [31], we used asparagine AAC in *Protaetia speciosa jousselini, Cyprolais quadrimaculata, Dicronorhina derbyana, Eudicella euthalia oweni, E. smithi, Goliathus goliatus, Jumnos ruckeri,* and *Mecynorhina torquata ugandensis* and AAT in *M. polyphemus.* The AAC and AAT were situated at 1 bp downstream of trnT. These two (AAC and AAT) start codons were hypothesized to be a synapomorphy for Coleoptera Polyphaga [31,42,43], and we confirmed this opinion. These two start codons have also been reported in Sericinae, Rutelinae, Melolonthinae [45], and even in Cetoniinae mt genomes [29,43]. The third atypical start codon found in our comparative study was GTC in Cetoniinae (*Cyprolais quadrimaculata)* and Dynastinae *(Dynastes hercules hercules)*, respectively, and was reported once in *Cheirotonus jansoni* (Scarabaeidae: Melolonthinae) [45]. In *C. quadrimaculata*, GTC was located 1 bp downstream of trnP to start ND6, whereas in *D. h. hercules*, it was adjacent to and downstream of trnM to start or initiate ND2. Their positions did not differ from those occupied by conventional start codons in the same position of these two genes. All the remaining PCGs started with the typical start codon.

### 4.4. Phylogenetic Relationship within Scarabaeidae

In all our Bayesian and maximum likelihood trees (Figure 3), we successfully recovered the monophyly of Rutelinae, Dynastinae, and Cetoniinae. All of our new sequenced and downloaded genomes of Dynastinae species constituted a monophylic clade and were recovered as a sister clade to Rutelinae. This sister relationship between Dynastinae and Rutelinae was also supported by some previous studies [6,19,81,82]. The nine new sequenced Cetoniinae species also clustered with the previously published Cetoniinae. Cetoniinae was found to be more closely related to Dynastinae and Rutelinae and their sister group relationship was strongly supported. In all our analyses, we failed to recover the monophyly of Melolonthinae and this result concurs with the previous published data for Melolonthinae [20,21]. The positioning of the second group of Melolonthinae (*Holotrichia* sp.) as a sister to (*C. jansoni + C. gestroi*) was not stable. This group of Melolonthinae was placed outside the main clade of the Melolonthinae comprising of ((*P. gracilicornis + P. l. mandshuricain*) + (*M. hippocastani + R. magnicornis*)), making Melolonthinae a non-monophyletic group. The placement of this group as a sister clade of Sericinae in both BI and ML trees using the AA dataset was considered based on AliGROOVE analyses as result of the high heterogeneity of this group. By contrast, with the PCG dataset, the subfamily of Sericinae species (*Pleophylla* sp. and *Serica* sp.) was placed as the most basal clade in this phytophagous group and a sister clade to all the phytophagous scarab lineages. This second relationship using the nucleotide dataset was highly supported compared with the first one (AA dataset), and our result was identical to that published by Song et al. [6]. From our present work, considering Sericinae which was monophyletic in all our analysis as a simple tribe Sericini of Melolonthinae was not strongly supported, so we agree with the proposal for the elevation of the Sericini tribe to subfamily level [6,20], and we also suggest that more taxa are needed to elucidate and confirm the phylogenic relationships within Sericinae.

## 5. Conclusions

In this study, we sequenced 18 mitochondrial genomes from scarab beetles belonging to Cetoniinae and Dynastinae for comparative molecular study and phylogenetic analysis. We discovered and described the particularity of the subfamily Dynastinae which presented a gene rearrangement of trnQ-NCR-trnI-trnM. This rearrangement of the cluster trnQ-NCR-trnI-trnM was found to be a synapomorphy in Dynastinae phytophagous scarabs. This region (CR to ND2) was designated as a hotspot region of gene rearrangement. The TDRL model was proposed to explain the mechanism of this rearrangement. We also found that the start codon asparagine (AAC and AAT) in COX1 genes was a synapomorphy in Cetoniinae (Coleoptera). These particular different characteristics help to support the monophyly of Cetoniinae and Dynastinae. The phylogenetic analyses recovered the monophyly of Rutelinae and the non-monophyly of Melolonthinae. Furthermore, our results support an elevation of the Sericini tribe to subfamily rank (Sericinae). This study reinforces our understanding of the identification of phytophagous scarab beetles and remind us of the importance of exploring the mitochondrial genome.

## Figures and Tables

**Table 1:** Information of the 34 species downloaded from GenBank used in the phylogenetic analyses.

Subfamily	Species	Accession No.	Size (bp)	Reference
Ingroups				
Cetoniinae	*Protaetia brevitarsis*	KC775706	20,319	[42]
	*Osmoderma opicum*	KU500641	15,341	[43]
Dynastinae	*Eophileurus chinensis*	MW632132	16,624	[18]
	*Oryctes rhinoceros*	MT457815	20,898	[19]
Rutelinae	*Popillia mutans*	MF997049	16,192	[6]
	*Popillia japonica*	NC_038115	16,541	[29]
	*Adoretus* sp.	JX412788	12,581	Unpublished
	*Mimela splendens*	MZ064554	15,148	Unpublished
Melolonthinae	*Polyphylla gracilicornis*	NC_054285	16,793	Unpublished
	*Cheirotonus gestroi*	MN893347	16,899	[44]
	*Melolontha hippocastani*	KX087316	15,485	Unpublished
	*Cheirotonus jansoni*	NC_023246	17,249	[45]
	*Rhopaea magnicornis*	NC_013252	17,522	[46]
	*Polyphylla laticollis mandshurica*	KF544959	14,473	[47]
	*Holotrichia oblita*	MF997046	15,968	[6]
Sericinae	*Pleophylla* sp.	JX412736	12,579	Unpublished
	*Serica* sp.	MF997050	13,815	[6]
Aphodiinae	*Aphodius foetens*	KX087240	15,907	Unpublished
Scarabaeinae	*Coprophanaeus* sp.	KU739465	15,554	[48]
	*Bubas bubalus*	KU739469	16,035	[48]
	*Onthophagus rhinolophus*	KU739498	15,237	[48]
	*Onthophagus vulpes*	KU739474	15,884	[48]
	*Scaptodera rhadamistus*	KU739460	15,119	[48]
	*Cheironitis hoplosternus*	KU739450	14,924	[48]
	*Euoniticellus intermedius*	KU739490	15,578	[48]
	*Liatongus militaris*	KU739488	15,832	[48]
	*Tiniocellus sarawacus*	KU739486	15,592	[48]
	*Caccobius nigritulus*	KU739484	15,039	[48]
	*Heteronitis castelnaui*	KU739468	13,441	[48]
	*Scarabaeidae* sp.	KT696268	18,626	Unpublished
Trogidae	*Omorgus chinensis*	MK937809	18,858	Unpublished
Glaresidae	*Glaresis* sp.	JX412819	12,663	Unpublished
Lucanidae	*Dorcus koreanus*	NC_054278	17,787	[49]
	*Lucanus* sp.	KT876903	20,634	[50]

**Table 2:** Comparative start and stop codons of Cetoniinae and Dynastinae mitochondrial genomes. Cetoniinae species designations are shaded: *Protaetia speciosa jousselini (P.s.j), Cyprolais quadrimaculata (C. q), Dicronorhina derbyana (D. d), Eudicella euthalia oweni (E e. o), E. smithi (E. s), Goliathus goliatus (G. g), Jumnos ruckeri (J.r.), Mecynorhina polyphemus, M. t. ugandensis (M.t. u)*. Dynastinae species designations are shaded: *Chalcosoma caucasus caucasus (C.c.c), Dynastes hercules hercules (D.h.h), Megasoma elephas elephas (M.e.e), M. mars (M.m), Oryctes nasicornis (O.n), Xylotrupes beckeri intermedius (X.b.i), X. g. beckeri (X.g.b), X. g. siamensis (X.g.s), X. g. sumatrensis (X.g.s).*

Species	Protein Coding Genes (PCGs) START-STOP CODONS												
	ND2	COX1	COX2	COX3	ATP8	ATP6	ND3	ND5	ND4	ND4L	ND6	CYTB	ND1
*P.s.j*	ATT/TAG	AAC/T	ATT/T	ATC/T	ATG/TAA	ATG/TAA	ATT/TAG	ATT/TAA	ATG/T	ATG/TAA	ATT/TAA	ATG/TAG	ATT/TAA
*C.q*	ATA/TAA	AAC/T	ATA/T	ATC/T	ATG/TAA	ATG/TAA	ATA/TAG	ATA/TAA	ATG/TAA	ATG/TAA	GTC/TAA	ATG/TAG	ATA/TAG
*D.d*	ATT/TAA	AAC/T	ATA/T	ATC/T	ATG/TAA	ATG/TAA	ATT/TAG	ATT/TAA	ATG/TAG	ATG/TAA	ATT/TAA	ATG/TAG	ATT/TAA
*E.e.o*	ATA/TAA	AAC/T	ATA/T	ATC/T	ATG/TAA	ATG/TAA	ATA/TAG	ATA/TAA	ATG/TAG	ATG/TAA	ATC/TAA	ATG/TAG	ATA/TAA
*E.s*	ATA/TAA	AAC/T	ATA/T	ATC/T	ATG/TAA	ATG/TAA	ATA/TAG	ATA/TAA	ATG/TAG	ATG/TAA	ATC/TAA	ATG/TAG	ATA/TAA
*G.g*	ATT/TAA	AAC/T	ATT/T	ATT/T	ATG/TAA	ATG/TAA	ATC/TAG	ATT/TAA	ATG/TAG	ATG/TAA	ATT/TAA	ATG/TAG	ATT/TAA
*J.r*	ATA/TAA	AAC/T	ATA/T	ATA/T	ATG/TAA	ATG/TAA	ATT/TAG	ATT/TAA	ATG/TAG	ATG/TAA	ATC/TAA	ATG/TAG	ATT/TAG
*M.p*	ATA/TAA	AAT/T	ATA/T	ATT/T	ATG/TAA	ATG/TAA	ATT/TAG	ATT/TAA	ATG/TAA	ATG/TAA	ATT/TAA	ATG/TAG	ATT/TAA
*M.t.u*	ATT/TAA	AAC/T	ATT/T	ATT/T	ATG/TAA	ATG/TAA	ATC/TAG	ATT/TAA	ATG/TAG	ATG/TAA	ATT/TAA	ATG/TAG	ATT/TAA
*C.c.c*	ATT/TAA	ATT/T	ATT/T	ATC/T	ATG/TAA	ATG/TAA	ATC/TAG	ATT/T	ATG/TAA	ATG/TAA	ATT/TAA	ATG/TAG	ATT/TAA
*D.h.h*	GTC/TAA	ATC/T	ATC/T	ATT/T	ATG/TAA	ATG/TAA	ATC/TAG	ATT/T	ATG/TAA	ATG/TAA	ATC/TAA	ATG/TAG	ATT/TAA
*M.e.e*	ATC/TAA	ATT/T	ATC/T	ATT/T	ATG/TAA	ATG/TAA	ATC/TAG	ATT/T	ATG/TAA	ATG/TAA	ATC/TAA	ATG/TAA	ATT/TAA
*M.m*	ATC/TAA	ATT/T	ATC/T	ATT/T	ATG/TAA	ATG/TAA	ATC/TAG	ATT/T	ATG/TAA	ATG/TAA	ATC/TAA	ATG/TAA	ATT/TAA
*O.n*	ATA/TAA	ATC/T	ATA/T	ATT/T	ATG/TAA	ATG/TAA	ATC/TAG	ATT/T	ATG/TAA	ATG/TAA	ATC/TAA	ATG/TAG	ATT/TAA
*X.b.i*	ATT/TAA	ATT/T	ATT/T	ATC/T	ATG/TAA	ATG/TAA	ATC/TAG	ATT/T	ATG/TAA	ATG/TAA	ATT/TAA	ATG/TAG	ATT/TAA
*X.g.b*	ATT/TAA	ATT/T	ATT/T	ATT/T	ATG/TAA	ATG/TAA	ATC/TAG	ATT/T	ATG/TAA	ATG/TAA	ATT/TAA	ATG/TAG	ATT/TAA
*X.g.s*	ATT/TAA	ATT/T	ATT/T	ATC/T	ATG/TAA	ATG/TAA	ATC/TAG	ATT/T	ATG/TAA	ATG/TAA	ATT/TAA	ATG/TAG	ATT/TAA
*X.g.s*	ATT/TAA	ATT/T	ATT/T	ATC/T	ATG/TAA	ATG/TAA	ATC/TAG	ATT/T	ATG/TAA	ATG/TAA	ATT/TAA	ATG/TAG	ATT/TAA

## Data Availability

The data supporting the findings of this study are openly available in National Center for Biotechnology Information at https://www.ncbi.nlm.nih.gov (accessed on 5 September 2021), accession numbers were: OK484299-OK484316.

## Supplementary Materials

The following are available online at www.mdpi.com/2075-4450/12/11/1025/s1. Figure S1. Circular maps of mitogenomes of Cetoniinae species (A): *Protaetia speciosa jousselini* (1), *Cyprolais quadrimaculata* (2), *Dicronorhina derbyana* (3), *Eudicella euthalia oweni* (4), *Eudicella smithi* (5), *Goliathus goliatus* (6), *Jumnos ruckeri* (7), *Mecynorhina polyphemus* (8), *Mecynorhina torquata ugandensis* (9). Circular maps of mitogenomes of Dynastinae species (B): *Chalcosoma caucasus caucasus* (1), *Dynastes hercules hercules* (2), *Oryctes nasicornis* (3), *Megasoma elephus elephus* (4), *Megasoma mars* (5), *Xylotrupes beckeri intermedius* (6), *Xylotrupes gideon beckeri* (7), *Xylotrupes gideon siamensis* (8), *Xylotrupes gideon sumatrensis* (9). Figure S2. Alignment of the intergenic spacer region located between ND1 and tRNA Ser (UCN) from all sequenced Cetoniinae (A) and Dynastinae (C) insects. (A) indicates the conserved hexanucleotide region (TACTAA) and (C) the Pentanucleotide region (TACTA) detected in all sequenced Cetoniinae and Dynastinae insects, respectively. (B) indicates the C-stretches and alignment of the intergenic spacer region located between trnK and trnD from all sequenced Dynastinae insects. Figure S3. The relative synonymous codon usage (RSCU) in each Cetoniinae and Dynastinae species mitogenomes. Codon families are provided on the *x*-axis and the different combinations of synonymous codons that code for each amino acid is defined on the *y*-axis. Table S1: The partition schemes and best-fitting models selected of 13 mitochondrial PCG and AA. Table S2. Location of features in the mitochondrial genome of Cetoniinae (A) and Dynastinae species (B). Table S3. Nucleotide composition and skewness of the whole mitogenomes of Cetoniinae and Dynastinae species. Table S4. Base composition, the percent of A, C, G and T, AT skew and GC skew of nucleotide sequence of 13PCGs of Cetoniinae and Dynastinae species. Table S5: NCR1 and NCR2 sequences in Dynastinae species. NCR = Non-coding Region. Table S6. Nucleotide composition and skewness of the A-T rich region of Cetoniinae and Dynastinae species. Table S7. Characteristics of the putative tandem repeats in the control region of Cetoniinae and Dynastinae species mitogenome. Table S8. Total number of codons, codon frequency and usage for each amino acid of mitochondrial genome of Cetoniinae and Dynastinae species.
