# Supplementary material for: The Mitochondrial Genomes of 18 New Pleurosticti (Coleoptera: Scarabaeidae) Exhibit a Novel trnQ-NCR-trnI-trnM Gene Rearrangement and Clarify Phylogenetic Relationships of Subfamilies within Scarabaeidae
Source: Insects. 2021 Nov 14;12(11):1025. doi: 10.3390/insects12111025 (PMC8622766; doi:10.3390/insects12111025)
Supplement: Supplementary file 1 [file Figure S2.pdf › Figure S2.pdf]

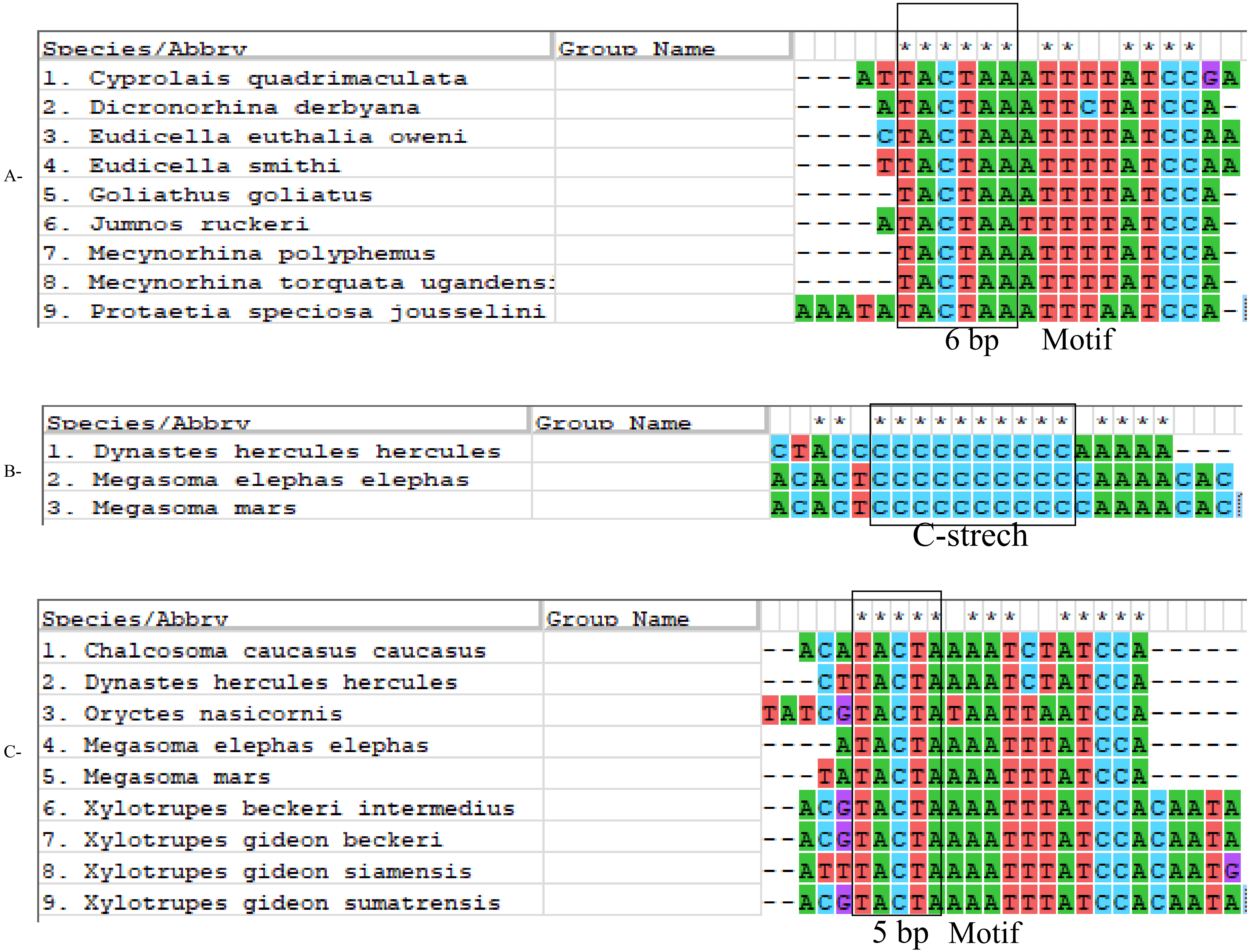

Figure S2. Alignment of the intergenic spacer region located between ND1 and tRNA Ser (UCN) from all sequenced Cetoniinae (A) and Dynastinae (C) insects. (A) indicate the conserved hexanucleotide region (TACTAA) and (C) the pentanucleotide region (TACTA) detected in all sequenced Cetoniinae and Dynastinae insects, respectively. (B) indicate the C-streches and alignment of the intergenic spacer region located between trnK and trnD from all sequenced Dynastinae (B) insects.
