# Supplementary material for: The Mitochondrial Genomes of 18 New Pleurosticti (Coleoptera: Scarabaeidae) Exhibit a Novel trnQ-NCR-trnI-trnM Gene Rearrangement and Clarify Phylogenetic Relationships of Subfamilies within Scarabaeidae
Source: Insects. 2021 Nov 14;12(11):1025. doi: 10.3390/insects12111025 (PMC8622766; doi:10.3390/insects12111025)
Supplement: Supplementary file 1 [file insects-12-01025-s001.zip › Figure S1.pdf]

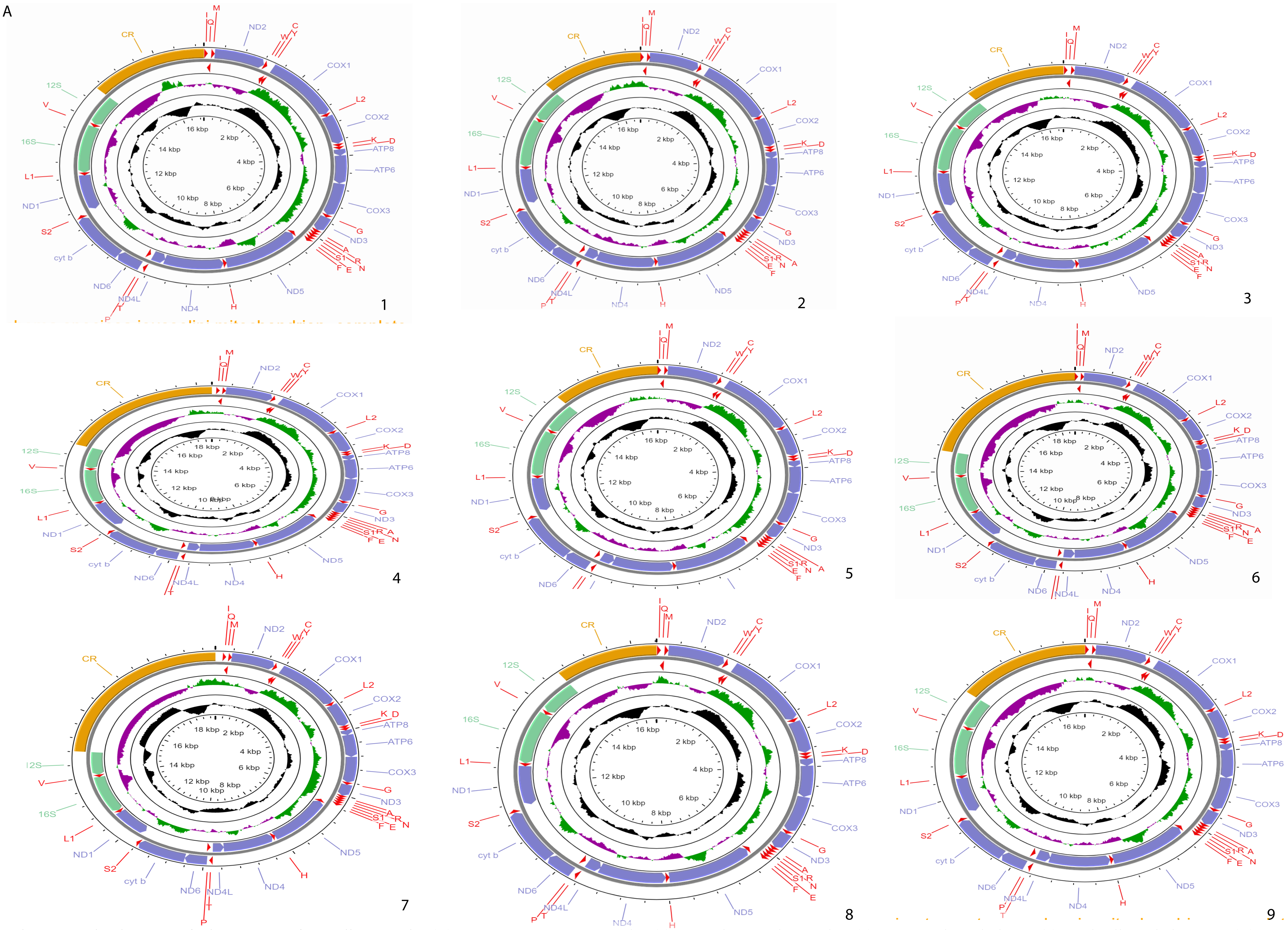

Figure S1. Circular maps of mitogenomes of Cetoniinae species (A): *Protaetia speciosa jousselini* (1), *Cyprolais quadrimaculata* (2), *Dicronorhina derbyana* (3), *Eudicella euthalia oweni* (4), *Eudicella smithi* (5), *Goliathus goliatus* (6), *Jumnos ruckeri* (7), *Mecynorhina polyphemus* (8), *Mecynorhina torquata ugandensis* (9).

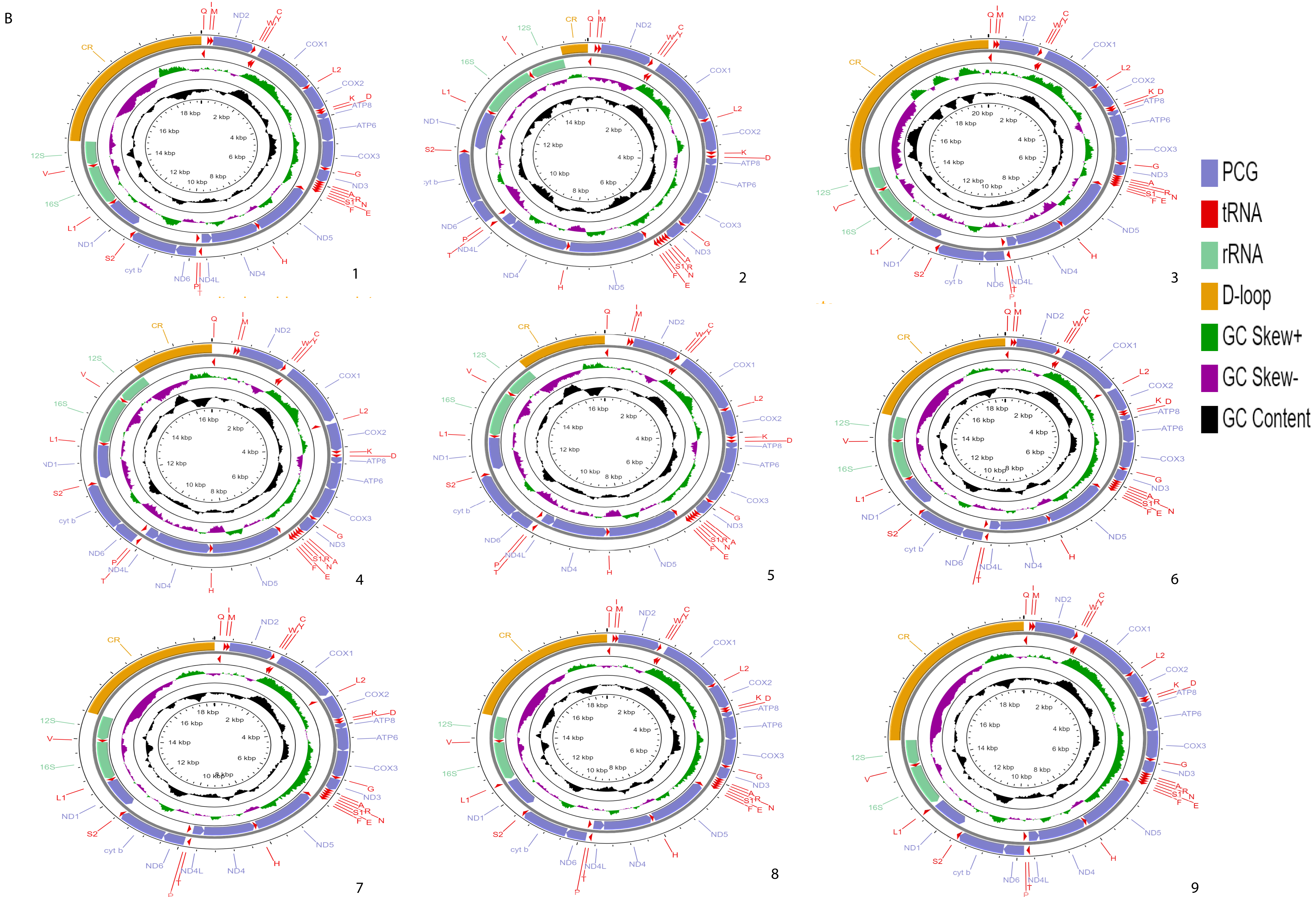

Figure S1. Circular maps of mitogenomes of Dynastinae species (B): *Chalcosoma Caucasus Caucasus* (1), *Dynastes hercules hercules* (2), *Oryctes nasicornis* (3), *Megasoma elephus elephus* (4), *Megasoma mars* (5), *Xylotrupes beckeri intermedius* (6), *Xylotrupes gideon beckeri* (7), *Xylotrupes gideon siamensis* (8), *Xylotrupes gideon sumatrensis* (9).
