# Supplementary material for: The Mitochondrial Genomes of 18 New Pleurosticti (Coleoptera: Scarabaeidae) Exhibit a Novel trnQ-NCR-trnI-trnM Gene Rearrangement and Clarify Phylogenetic Relationships of Subfamilies within Scarabaeidae
Source: Insects. 2021 Nov 14;12(11):1025. doi: 10.3390/insects12111025 (PMC8622766; doi:10.3390/insects12111025)
Supplement: Supplementary file 1 [file insects-12-01025-s001.zip › Figure S3.pdf]

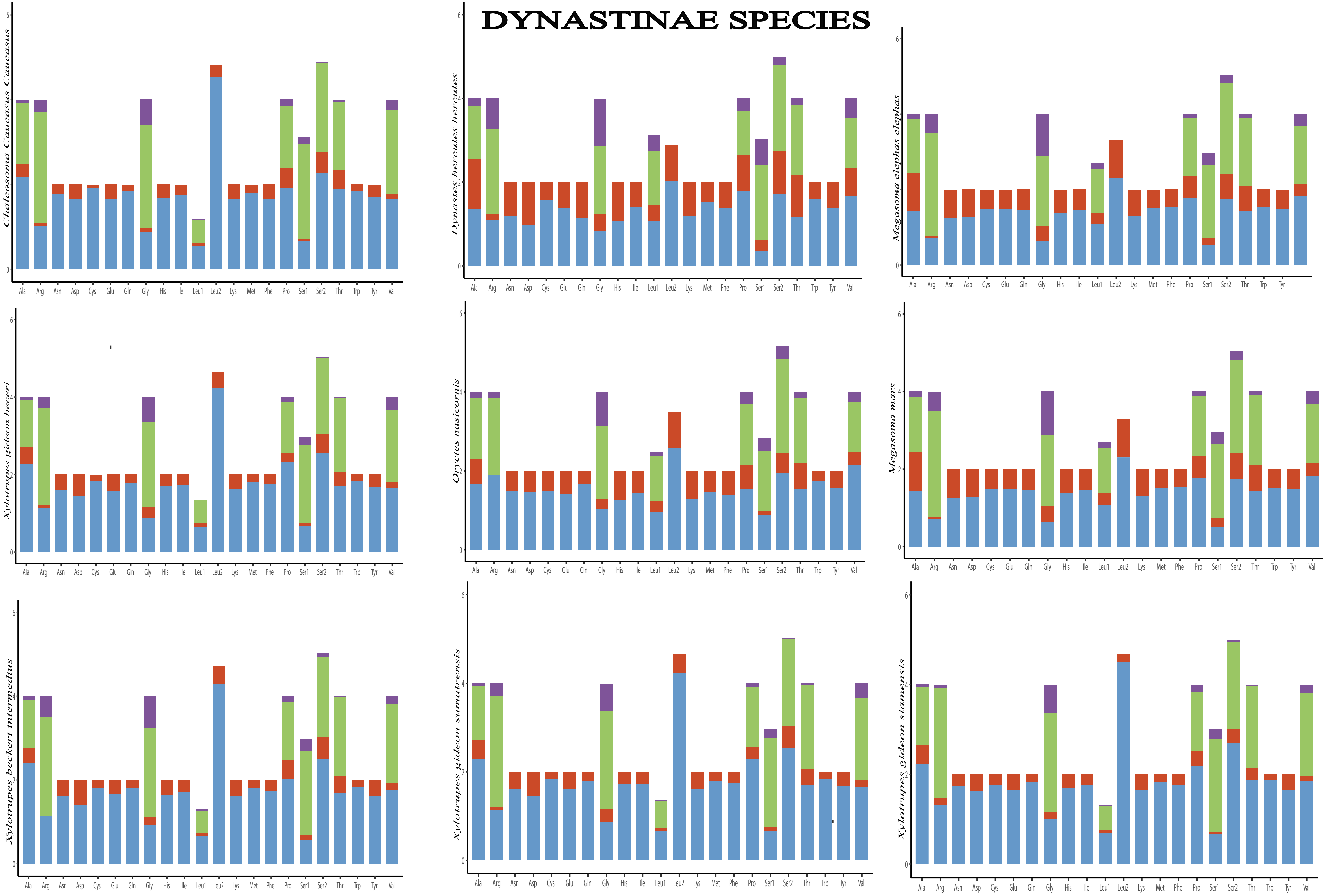
DYNASTINAE SPECIES
Chalcosoma Caucasus Caucasus
Dynastes hercules hercules
Megasoma elephas elephas
Xylotrupes gideon beceri
Oryctes nasicornis
Megasoma mars
Xylotrupes beckeri intermedius
Xylotrupes gideon sumatrensis
Xylotrupes gideon siamensis
Ala Arg Asn Asp Cys Glu Gln Gly His Ile Leu1 Leu2 Lys Met Phe Pro Ser1 Ser2 Thr Trp Tyr Val

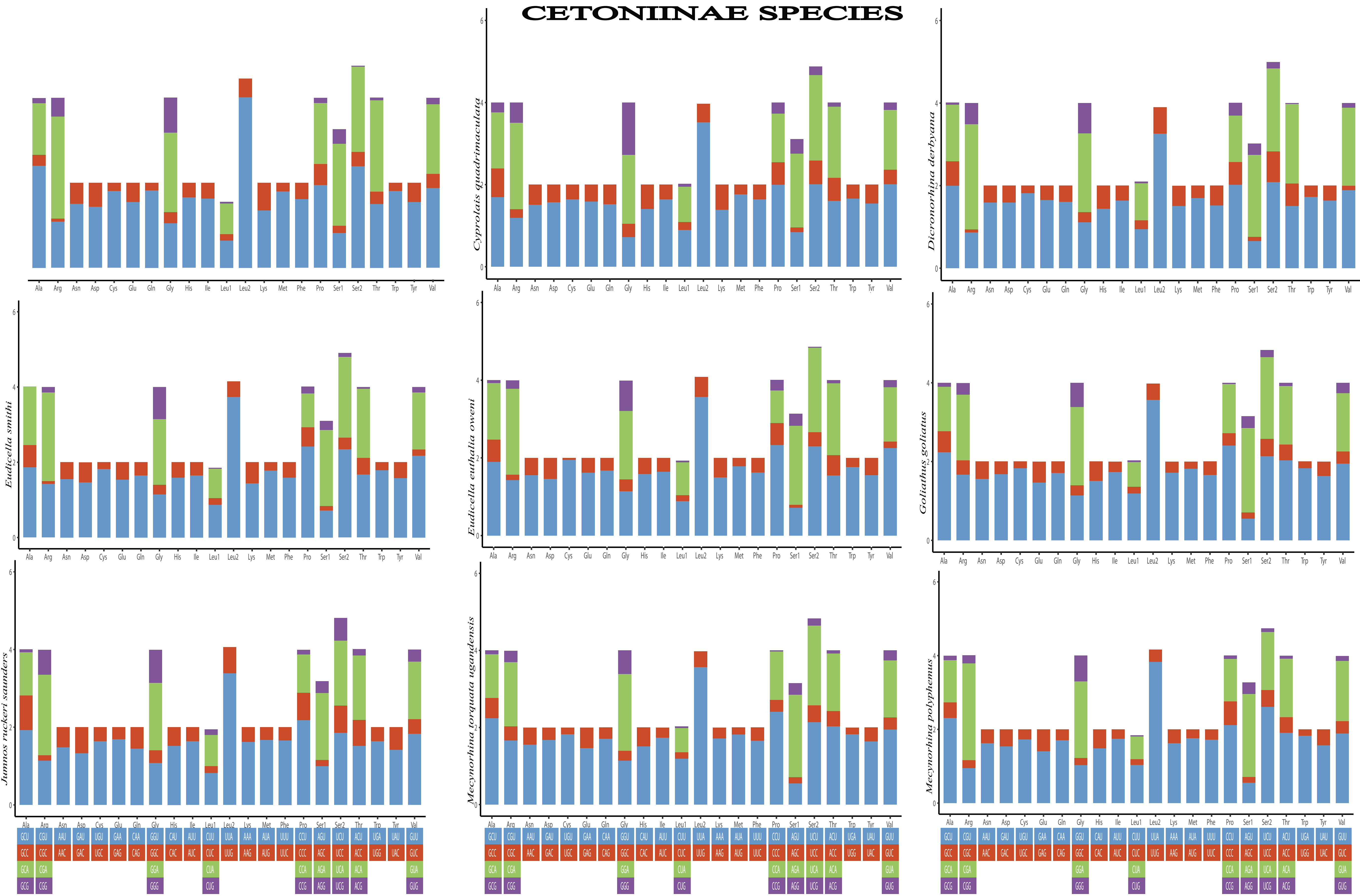

Figure S3. The relative synonymous codon usage (RSCU) in each Cetoniinae and Dynastinae species mitogenomes. Codon families are provided on the x-axis and the different combinations of synonymous codons that code for each amino acid is defined on the y-axis
