# Supplementary material for: The Mitochondrial Genomes of 18 New Pleurosticti (Coleoptera: Scarabaeidae) Exhibit a Novel trnQ-NCR-trnI-trnM Gene Rearrangement and Clarify Phylogenetic Relationships of Subfamilies within Scarabaeidae
Source: Insects. 2021 Nov 14;12(11):1025. doi: 10.3390/insects12111025 (PMC8622766; doi:10.3390/insects12111025)
Supplement: Supplementary file 1 [file insects-12-01025-s001.zip › Table S1.pdf]

Table S1: The partition schemes and best-fitting models selected of 13 mitochondrial PCGs and AAs.

A. Nucleotide sequence alignements

Subset	Best model	Subset partitions
Partition1	GTR+I+G	cox2_codon1, cox3_codon1, cytb_codon1, atp6_codon1
Partition2	TVM+I+G	cox2_codon2, cytb_codon2, atp6_codon2, cox1_codon2, cox3_codon2
Partition3	TRN+I+G	nad3_codon3, atp6_codon3, atp8_codon3, nad6_codon3
Partition4	GTR+I+G	atp8_codon1, nad2_codon1, nad6_codon1, nad3_codon1
Partition5	TVM+I+G	nad3_codon2, nad2_codon2, nad6_codon2, atp8_codon2
Partition6	SYM+G	cox1_codon1
Partition7	TVM+G	cytb_codon3, cox3_codon3, cox2_codon3, cox1_codon3
Partition8	GTR+I+G	nad4L_codon1, nad4_codon1, nad5_codon1, nad1_codon1
Partition9	GTR+I+G	nad4L_codon2, nad1_codon2, nad4_codon2, nad5_codon2
Partition10	GTR+I+G	nad4_codon3, nad1_codon3, nad5_codon3, nad4L_codon3
Partition11	HKY+G	nad2_codon3

B. Amino Acid sequence alignements

Subset	Best model	Subset partitions
Partition1	MTART+I+G	cox3, atp6, cytb
Partition2	MTREV+I+G+F	atp8, nad3, nad2, nad6
Partition3	MTART+I+G	cox1
Partition4	MTMAM+I+G	cox2
Partition5	MTART+I+G	nad5, nad1
Partition6	MTART+I+G	nad4, nad4L
