# Supplementary material for: The Mitochondrial Genomes of 18 New Pleurosticti (Coleoptera: Scarabaeidae) Exhibit a Novel trnQ-NCR-trnI-trnM Gene Rearrangement and Clarify Phylogenetic Relationships of Subfamilies within Scarabaeidae
Source: Insects. 2021 Nov 14;12(11):1025. doi: 10.3390/insects12111025 (PMC8622766; doi:10.3390/insects12111025)
Supplement: Supplementary file 1 [file insects-12-01025-s001.zip › Table S7.pdf]

Table S7. Characteristics of the putative tandem repeats in the control region of Cetoniinae and Dynastinae species mitogenome.

Table Explanation

Protaetia speciosa jousselini

Indices	Period Size	Copy Number	Consensus Size	Percent Matches	Percent Indels	Score	A	C	G	T	Entropy (0-2)
31--94	13	4.9	14	76	19	50	51	3	4	40	1.38
222--462	119	2.0	119	96	0	448	42	22	4	29	1.73
729--1013	83	3.4	83	99	0	561	35	16	4	43	1.70
1355--1418	2	33.0	2	75	12	53	43	1	4	50	1.32
1652--1712	25	2.4	25	78	15	72	52	4	0	42	1.23
1742--1772	16	1.9	16	93	0	53	48	3	0	48	1.17

Table Explanation

Cyprolais quadrimaculata

Indices	Period Size	Copy Number	Consensus Size	Percent Matches	Percent Indels	Score	A	C	G	T	Entropy (0-2)
572--777	79	2.6	79	100	0	412	33	12	4	48	1.62
861--909	25	1.9	26	84	12	66	42	2	4	51	1.32
1103--1134	14	2.4	14	90	10	50	34	0	0	65	0.93
1402--1467	17	3.8	17	75	15	55	56	0	3	40	1.15
1489--1537	21	2.4	20	87	12	73	53	2	6	38	1.38

Table Explanation

Dicronorhina derbyana

Indices	Period Size	Copy Number	Consensus Size	Percent Matches	Percent Indels	Score	A	C	G	T	Entropy (0-2)
572--777	79	2.6	79	100	0	412	33	12	4	48	1.62
861--909	25	1.9	26	84	12	66	42	2	4	51	1.32
1103--1134	14	2.4	14	90	10	50	34	0	0	65	0.93
1402--1467	17	3.8	17	75	15	55	56	0	3	40	1.15
1489--1537	21	2.4	20	87	12	73	53	2	6	38	1.38

WPS PDF编辑试用

Table Explanation

Eudicella euthalia oweni

Indices	Period Size	Copy Number	Consensus Size	Percent Matches	Percent Indels	Score	A	C	G	T	Entropy (0-2)
247--276	2	15.0	2	100	0	60	50	0	0	50	1.00
323--1999	259	6.5	259	97	0	2899	46	17	4	29	1.67
2669--2705	13	2.6	15	87	8	51	45	2	0	51	1.15
3016--3077	18	3.5	18	77	14	58	56	0	0	43	0.99
3037--3077	8	4.7	9	79	14	50	53	0	0	46	1.00
3035--3074	10	4.2	10	76	23	50	52	0	0	47	1.00
3037--3074	20	1.9	20	94	0	67	52	0	0	47	1.00
3120--3152	12	2.5	14	85	14	52	48	0	0	51	1.00

Table Explanation

Eudicella smithi

Indices	Period Size	Copy Number	Consensus Size	Percent Matches	Percent Indels	Score	A	C	G	T	Entropy (0-2)
1429--1490	18	3.5	18	77	14	58	56	0	0	43	0.99
1450--1490	8	4.7	9	79	14	50	53	0	0	46	1.00
1448--1487	10	4.2	10	76	23	50	52	0	0	47	1.00
1450--1487	20	1.9	20	94	0	67	52	0	0	47	1.00

Table Explanation

Goliathus goliatus

Indices	Period Size	Copy Number	Consensus Size	Percent Matches	Percent Indels	Score	A	C	G	T	Entropy (0-2)
264--551	93	3.1	93	93	2	481	38	26	3	31	1.73
430--1177	313	2.4	313	98	0	1433	40	18	5	35	1.73
1056--1363	93	3.3	93	94	0	546	37	23	5	32	1.78
1555--2048	93	5.3	93	94	0	873	38	25	4	31	1.75
743--1862	499	2.2	499	95	0	2046	39	20	5	34	1.75
2240--2436	91	2.1	93	93	1	335	38	24	5	31	1.77
2784--2818	18	1.9	18	88	0	52	37	8	0	54	1.31
3190--3294	23	4.6	23	71	17	60	48	1	0	49	1.12
3186--3269	19	4.6	19	69	18	54	47	2	0	50	1.14

Table Explanation

Jumnos ruckeri

Indices	Period Size	Copy Number	Consensus Size	Percent Matches	Percent Indels	Score	A	C	G	T	Entropy (0-2)
213--265	22	2.4	22	93	3	88	60	9	1	28	1.38
340--2058	243	7.1	243	99	0	3357	34	28	8	28	1.87
2111--3165	86	12.3	86	95	0	1772	34	28	9	26	1.88
3388--3514	40	3.2	40	63	14	107	35	17	5	41	1.73
3401--3528	41	3.1	41	63	14	111	33	17	6	42	1.75
3355--3570	81	2.7	81	100	0	432	33	13	6	46	1.69

WPS PDF编辑试用

Table Explanation

Mecynorhina Polyphemus

Indices	Period Size	Copy Number	Consensus Size	Percent Matches	Percent Indels	Score	A	C	G	T	Entropy (0-2)
297--546	81	3.1	81	92	5	418	39	14	3	43	1.61
920--1094	46	3.8	45	68	18	119	48	2	0	49	1.18
964--1088	27	4.3	29	67	20	73	48	2	0	48	1.20
1174--1218	21	2.0	21	83	12	54	60	0	4	35	1.17
1168--1220	22	2.5	20	76	14	52	58	0	3	37	1.16

Table Explanation

Mecynorhina torquata ugandensis

Indices	Period Size	Copy Number	Consensus Size	Percent Matches	Percent Indels	Score	A	C	G	T	Entropy (0-2)
264--551	93	3.1	93	93	2	481	38	26	3	31	1.73
430--1178	313	2.4	313	98	0	1435	40	18	5	35	1.73
1277--1311	18	1.9	18	88	0	52	37	8	0	54	1.31
1683--1787	23	4.6	23	71	17	60	48	1	0	49	1.12
1679--1762	19	4.6	19	69	18	54	47	2	0	50	1.14

Table Explanation

Chalcosoma caucasus caucasus

Indices	Period Size	Copy Number	Consensus Size	Percent Matches	Percent Indels	Score	A	C	G	T	Entropy (0-2)
107--1283	82	14.4	82	95	0	1886	42	16	8	29	1.77
1283--1717	125	3.5	125	97	0	825	39	23	1	35	1.66
3331--3375	10	4.4	10	83	5	54	51	11	11	26	1.71
3648--3689	21	2.0	22	85	4	59	42	2	0	54	1.13
3659--3760	21	4.8	22	76	21	87	44	2	0	51	1.23
3770--3820	25	2.0	25	84	7	66	47	5	1	45	1.38
3964--4064	15	6.8	14	67	15	51	45	0	2	51	1.16

Table Explanation

Dynastes hercules hercules

Indices	Period Size	Copy Number	Consensus Size	Percent Matches	Percent Indels	Score	A	C	G	T	Entropy (0-2)
90--1181	72	15.2	72	98	0	1788	21	23	18	31	1.95

WPS PDF编辑试用

Table Explanation

Megasoma elephas elephas

Indices	Period Size	Copy Number	Consensus Size	Percent Matches	Percent Indels	Score	A	C	G	T	Entropy (0-2)
180--713	198	2.7	198	99	0	1059	33	29	11	26	1.90

Table Explanation

Megosoma mars

Indices	Period Size	Copy Number	Consensus Size	Percent Matches	Percent Indels	Score	A	C	G	T	Entropy (0-2)
183--914	198	3.7	198	99	0	1437	33	29	11	25	1.90

Table Explanation

Oryctes nasicornis

Indices	Period Size	Copy Number	Consensus Size	Percent Matches	Percent Indels	Score	A	C	G	T	Entropy (0-2)
535--887	186	1.9	186	96	0	652	43	24	6	25	1.79
861--1733	273	3.2	273	99	0	1692	29	31	11	26	1.91
1747--2574	186	4.5	186	99	0	1602	43	23	7	25	1.79
2788--2845	12	4.4	13	77	18	50	50	3	0	46	1.18
2788--2844	14	4.5	13	77	14	57	49	3	0	47	1.18
2787--2842	26	2.3	23	82	14	60	50	3	0	46	1.19
3697--3754	12	4.4	13	77	18	50	50	3	0	46	1.18
3697--3753	14	4.5	13	77	14	57	49	3	0	47	1.18
3696--3751	26	2.3	23	82	14	60	50	3	0	46	1.19
4606--4663	12	4.4	13	77	18	50	50	3	0	46	1.18
4606--4662	14	4.5	13	77	14	57	49	3	0	47	1.18
4605--4660	26	2.3	23	82	14	60	50	3	0	46	1.19

Table Explanation

Xylotrupes beckeri intermedius

Indices	Period Size	Copy Number	Consensus Size	Percent Matches	Percent Indels	Score	A	C	G	T	Entropy (0-2)
257--1395	124	9.2	124	84	3	1045	39	18	3	38	1.67
687--1452	374	2.0	371	97	0	1433	38	21	3	36	1.70
687--1452	125	6.2	125	86	3	1046	38	21	3	36	1.70
2820--2868	10	4.9	10	75	24	50	46	2	0	51	1.12
2819--2857	12	3.3	12	82	13	53	48	2	0	48	1.15
3127--3235	46	2.3	48	77	15	118	49	2	0	47	1.15
3155--3245	46	2.0	46	80	8	105	48	2	1	48	1.21

Table Explanation

Xylotrupes g. siamensis

Indices	Period Size	Copy Number	Consensus Size	Percent Matches	Percent Indels	Score	A	C	G	T	Entropy (0-2)
227--836	124	4.9	124	99	0	1175	41	14	1	42	1.55
1094--1385	125	2.3	125	95	0	521	38	23	2	34	1.69
1283--1610	175	1.9	174	88	3	498	38	20	2	39	1.65
1872--1926	20	2.9	19	75	25	55	43	7	1	47	1.41
2329--2385	29	1.9	30	82	10	73	40	12	3	43	1.59
2743--2803	32	1.9	32	82	0	77	47	11	6	34	1.66
2900--2945	22	2.1	21	88	4	65	43	2	2	52	1.25
3251--3306	29	2.0	27	82	6	67	48	3	0	46	1.19

WPS PDF

Table Explanation

Xylotrupes gideon beckeri

Indices	Period Size	Copy Number	Consensus Size	Percent Matches	Percent Indels	Score	A	C	G	T	Entropy (0-2)
218--1262	126	8.3	126	88	3	1405	40	22	2	34	1.66
218--1262	376	2.8	377	92	1	1668	40	22	2	34	1.66
1105--1149	21	2.0	23	83	8	58	40	17	6	35	1.76
2692--2807	22	5.3	21	68	26	73	48	1	1	48	1.22
2692--2802	12	9.0	12	66	23	61	47	1	1	48	1.22
2669--2789	64	1.9	64	81	9	151	45	2	3	48	1.32
3019--3094	15	5.1	15	65	16	50	44	0	2	52	1.14

Table Explanation

Xylotrupes g. sumatrensis

Indices	Period Size	Copy Number	Consensus Size	Percent Matches	Percent Indels	Score	A	C	G	T	Entropy (0-2)
221--2143	126	15.3	126	88	3	2345	40	22	2	35	1.67
304--2575	125	18.1	125	88	3	2123	39	22	2	35	1.69
3948--4063	22	5.3	21	68	26	73	48	1	1	48	1.22
3948--4058	12	9.0	12	66	23	61	47	1	1	48	1.22
3925--4045	64	1.9	64	81	9	151	45	2	3	48	1.32
